# Osteosarcoma Across the Age Spectrum: Why Outcomes Diverge and Trials Must Adapt

**DOI:** 10.3390/jcm15187017

**Published:** 2026-09-10

**Authors:** Samantha H. Collins, Igor Matushansky

**Affiliations:** Department of Medicine, Columbia University Medical Center, New York, NY 10032, USA; sc5729@cumc.columbia.edu

**Keywords:** osteosarcoma, adolescent and young adult oncology, pediatric oncology, sarcoma

## Abstract

Osteosarcoma clinical presentation, treatment tolerance, and treatment delivery differ across the age spectrum. Adult cohorts are more heterogeneous, with higher proportions of axial and secondary osteosarcoma and greater variation in comorbidity and treatment intensity. Furthermore, emerging genomic studies suggest age-associated differences in tumor complexity and stress-response pathways, whereas direct age-stratified epigenomic and immune-microenvironment data remain limited. These molecular observations are hypothesis-generating and data currently do not support age-specific treatment selection. This narrative review summarizes clinical and biological evidence across age groups, distinguishes prognostic associations from predictive treatment effects, and identifies major confounders and evidence gaps. The available evidence supports age-inclusive prospective trials that model age explicitly, incorporate biomarker-stratified analyses, and report delivered dose intensity, toxicity, and host factors.

## 1. Methods

We conducted a narrative literature search of PubMed/MEDLINE from database inception to 26 July 2026. The principal search concepts combined “osteosarcoma” with “age,” “pediatric,” “adolescent and young adult,” “older adult,” “elderly,” “survival,” “genomics,” “epigenetics,” “tumor microenvironment,” “immunotherapy,” “targeted therapy,” and “clinical trial.” We considered English-language peer-reviewed articles, relevant conference abstracts, clinical-trial records, and major clinical guidance, and we examined the reference lists of key reviews and eligible articles. Priority was ascribed to large population-based or cooperative-group cohorts, studies directly comparing age groups, osteosarcoma-specific molecular studies, and prospective clinical evidence. The evidence was synthesized narratively; study screening and risk-of-bias assessment were not performed under a formal systematic-review protocol.

Age terms are used descriptively and consistently unless a cited study imposed different boundaries: pediatric, <15 years; adolescent and young adult (AYA), 15–39 years; middle-aged adult, 40–59 years; and older adult, ≥60 years. Study-specific cutoffs are reported explicitly and should not be assumed to be biologically equivalent. Consistent with current World Health Organization terminology, “osteosarcoma” refers to high-grade conventional primary (non-secondary) osteosarcoma unless stated otherwise. Secondary osteosarcoma is included only where it materially contributes to an age-related pattern and is identified explicitly. Comparisons across age groups require caution because age is correlated with tumor site, stage, histologic subtype, primary versus secondary disease, technical resectability and margin status, comorbidity, treatment intensity, locus of care, and treatment era; each can confound an apparent effect of chronological age.

## 2. Introduction

Osteosarcoma (OS) is the most common primary bone cancer; however, it is still rare, with an estimated 800–900 cases diagnosed annually [1]. It can occur in any bone of the body, with the most common presentation in the metaphysis of the long bones, including the distal femur, proximal tibia, and proximal humerus [2,3]. OS has a bimodal age distribution with a peak incidence occurring during adolescence with a smaller second peak later in adulthood [1,2,4].

Age has long been understood to be one of the most powerful prognostic factors in osteosarcoma. Numerous studies have been conducted, assessing for differences in outcomes ranging from overall survival, relative survival, and disease-specific survival, among different age groups across numerous different geographic cohorts. In their analysis of >1700 newly diagnosed patients with OS registered into the Cooperative OS Study Group before 1998, Bielack et al. showed that patients over the age of 40 years carried 10-year overall survival rates of only 41.6% compared to 60.2% in those less than 40 years old [5]. It is important to acknowledge, however, that any differences in overall survival may be conflated by non-cancer-related mortality and comorbidity. Cole et al. analyzed osteosarcoma relative survival rates by age group with their analysis of the >5000 cases of osteosarcoma in the Surveillance, Epidemiology and End Results (SEER) program from 1975 to 2017 [2]. They showed that patients aged 0–9 years had a 5-year relative survival (RS) of 71.8%, patients aged 10–24 years had a 5-year RS of 65.9%, patients aged 25–59 years had 5-year RS 56.8%, and patients > 60 years old had a 5-year RS of 33.1%. While they assessed different age groups in their analysis, a recent cohort study conducted in Denmark of patients under the age of 40 years diagnosed with osteosarcoma showed that patients aged 0–14 years carried a 5-year RS of 60% and those aged 15–24 years had 56% [6], comparable to rates seen by Cole et al. [2]. Ogura et al. recently published results from their retrospective cohort analysis of >3000 patients diagnosed with osteosarcoma as part of Japan’s Bone and Soft Tissue Tumor Registry and noted 5-year disease-specific survival (DSS) rates of 66% overall, but noted a significant decline in disease-specific survival as age progressed when comparing patients of ages 0–14, 15–29, 40–59, 60–74, and 75+ years [4]. In their analysis, hazard ratios for DSS ranged from 1.04 in the adolescent and young adult (AYA) population (15–39 years old) to 2.35 in the elderly (60–74 years old) and 4.48 in the super elderly (75+) when compared to the pediatric population (aged <14 years).

While age group and outcomes assessed differ study to study, the conclusions remain consistent that younger patients consistently carry improved outcomes as compared to their older counterparts. Many hypotheses have been made to explain such differences, ranging from differences in treatment delivery, tumor location and stage, as well as tumor biology, which continue to evolve over time [7].

## 3. Disease Presentation

For patients aged 10–30 years, the majority of cases of OS occur in the appendicular skeleton with most cases occurring in the lower long bones [1,2]. While appendicular tumors are still the predominant lesions in adulthood, there is a higher incidence of axial skeleton lesions, including the pelvis, mandible, or skull [2].

In adults, axial tumors may be more common due to the relative increase in incidence of secondary osteosarcoma among the elderly with history of prior radiation exposure and Paget’s disease. Paget’s disease-associated osteosarcoma is rare, occurring in just 1% of cases of osteosarcoma in the SEER program of 2000–2017; however, Paget’s was more commonly seen in the elderly at 4.7% of the cases for patients > 60 y old [2]. Paget’s carries a poor prognosis historically with a 5-year overall survival of only 5–10% [8,9]. In the SEER program from 1975 to 2017, secondary osteosarcoma occurred most often following treatment for breast and prostate cancers in the elderly, compared to rhabdomyosarcoma and tumors of the central nervous system among younger patients [2]. The incidence of secondary osteosarcoma was the highest among the elderly, steadily increasing over the past few decades to being 1.9 per million in the most recent decade [2]. The 5-year relative survival rates for secondary osteosarcoma were shown to decline with age at 44% for 10–24-year-olds to 19% among patients >60 years old [2].

Histologic composition also varies with age. In a 438-patient cohort divided at 21 years, osteoblastic tumors were more frequent in younger patients (44.9% vs. 31.8%), whereas chondroblastic (18.9% vs. 13.1%), fibroblastic (19.6% vs. 15.9%), and mixed or other tumors were relatively more frequent in adults [10]. In 570 patients from two European Osteosarcoma Intergroup trials, histologic subtype was associated with chemotherapy response: fibroblastic tumors had a higher and chondroblastic tumors a lower frequency of good histologic response, but subtype did not significantly stratify overall survival [11]. Subtype distribution may contribute to age-associated outcome patterns, but it should not be treated as an independent explanation without adjustment for site, stage, secondary disease, resectability, treatment, and histologic response.

Approximately 10–15% of patients with newly diagnosed osteosarcoma present with metastatic disease [1]. In Cole et al.’s analysis of the SEER program, metastatic osteosarcoma was more common in the elderly (>60 years old) with 33% of the cases [2]. The observed frequency of metastatic disease varied by tumor location and was the greatest for osteosarcoma to the pelvis (41.7% of all cases) compared to other sites for all ages [2]. In their analysis, survival with metastatic disease also decreased with increasing age, with the youngest patients (0–24 years old) having 5-year relative survival of 35.5% compared to that of the elderly (>60 years old) only being 6.4% [2].

While these clear differences in disease presentation seem to drive the observed differences in outcomes between pediatric and adult osteosarcoma patients, a few studies’ attempts to account for these factors indicate a driving factor beyond tumor location and stage to explain the difference in outcomes. Boyland et al. assessed for differences in overall survival between patients aged 1–17 years and 18 years and older [12]. Following their multivariable analysis of over 8000 patients in the National Cancer Database, they showed the difference in overall survival persisted despite cancer stage and treatment modality. Janeway et al. showed that age 18 years or older was associated with a statistically significant poorer event-free survival (EFS) and overall survival and claim this difference was not explained by tumor location, metastatic disease, or histologic response following their multivariate analysis [13].

## 4. Treatment and Locus of Care

### 4.1. Time to Treatment Initiation

Han et al. conducted a SEER database analysis on patients diagnosed with osteosarcoma in 2000–2021 and looked at time to treatment initiation [14]. They state that there was improved survival for patients treated within one month of diagnoses as compared to those treated later and that delayed time to treatment initiation was more often seen among patients aged 10 years or older. Sasi et al. conducted a retrospective single-institutional study on patients with high-grade bone sarcomas from 2003 and 2018 [15]. Of the 1227 patients they analyzed, age > 18 years and tumor size above 7.5 cm, among other factors, were predictors of a longer diagnostic interval (>4 months). They, however, did not find the length of diagnostic interval significantly impacting amputation requirements or survival outcomes [15].

### 4.2. Surgical Resection and Reconstruction

The mainstay of osteosarcoma treatment consists of complete resection in combination with perioperative chemotherapy. Axial tumors have worse outcomes compared to appendicular tumors [2,5,16], hypothesized to be driven by increased technical difficulty of surgical resection [17], and possible delays in detection while occurring in the trunk. Breden et al. conducted a retrospective single center study among patients with high-grade osteosarcoma and revealed a significantly higher recurrence rate in patients with margins < 1 mm compared to those with wider margins [18]. The use of radiation therapy for osteosarcoma has been largely limited given the relative radio-resistance of OS. More recently, particle therapy has been explored in the treatment of unresectable [19], axial [20], and craniofacial [21] osteosarcoma; however, its role remains investigational.

Current reconstruction strategies include biological approaches such as allograft transplantation and endoprosthetic reconstruction [22,23,24,25]. Biological approaches may preserve native bone and growth potential in skeletally immature patients but require prolonged weight-bearing restrictions and carry risks of nonunion, graft failure, infection, and fracture. Endoprostheses provide immediate mechanical stability and earlier mobilization but may require later revision for infection or mechanical failure. In selected extremity or thigh sarcomas that involve major vessels, en bloc vascular resection with arterial and/or venous reconstruction can permit limb preservation and oncologically adequate margins [26,27]. These procedures require coordinated planning among orthopedic oncology, vascular surgery, plastic surgery, and medical oncology and carry graft, wound, thrombotic, and functional morbidity. The supporting literature is derived largely from soft-tissue sarcoma rather than osteosarcoma and is not age-stratified; it therefore informs surgical feasibility rather than an age-specific survival effect. A recent systematic review of more than 12,000 patients found that limb-salvage surgery was more frequent in pediatric than adult osteosarcoma, whereas adults had higher amputation and revision rates and lower rates of return to ambulation [28].

### 4.3. Tolerance of Systemic Treatment

The MAP regimen, consisting of high-dose methotrexate (HDMTX), doxorubicin, and cisplatin, has long been established as the systemic therapy of choice for osteosarcoma [29,30]. The MAP regimen carries a high level of toxicity, thought widely to be challenging to tolerate, with particular concern directed specifically to the elderly [17]. Multiple cohort analyses have shown that adults receive less chemotherapy overall than their pediatric and AYA counterparts due to dose reductions or therapy cessation as a result of or in anticipation of MAP-associated toxicities [4,31,32,33]. Abrahão et al. showed that there was age-related variation in the proportion of patients who received guideline-concordant care (GCC) after analysis of patients with OS in the California Cancer Registry of 2004–2019, with a reduction in GCC with rising age [34].

Doxorubicin carries a risk of cardiotoxicity, with complications including congestive heart failure and cardiomyopathy that carry a poor prognosis and high mortality risk [35]. Beyond the clear association between the development of heart failure and doxorubicin dose, multiple studies have showed that the risk of doxorubicin-associated heart failure increases with age [36,37]. Elderly patients are thought to be more susceptible to cardiotoxicity as a result of underlying cardiac comorbidities and limited cardiac reserve [38]. Interestingly, in a single center retrospective cohort study in the Netherlands of 528 patients diagnosed and treated with OS or Ewing sarcoma of 1983–2018, Heemelaar et al. showed that, while higher cumulative doxorubicin dose was associated with higher rates of heart failure, it was also associated with a slightly lower all-cause death, hypothesized to be related to patients having received complete doxorubicin treatment [37].

Cisplatin similarly carries age-associated toxicity risk with associations with nephrotoxicity, ototoxicity, neuropathy, and myelosuppression [39]. As cisplatin is renally cleared, a hypothesized mechanism for an increased toxicity risk among the elderly includes decreased clearance due to age-related reductions in renal function [40,41]. It is also thought that cisplatin likely exacerbates any age-related hearing loss as well as pre-existing neuropathies [39]. While such complications may not have implications on survival, such toxicities may carry a high burden on patient quality of life.

Finally, HDMTX is associated with acute kidney injury (AKI) as a result of tubular obstruction related to the crystallization of methotrexate and its metabolites within the lumen as well as direct toxicity on tubular cells [42]. Methotrexate is cleared renally [43]; so, any preceding or methotrexate-induced renal dysfunction results in delayed methotrexate excretion and sustained elevated plasma concentrations, increasing the risk for additional toxicities of methotrexate, including myelosuppression and hepatotoxicity [42,44]. Delayed clearance of methotrexate has been shown to significantly correlate with age [45].

Due to fear of high toxicity levels, it is not uncommon for the use of adjuvant high dose methotrexate to be omitted with older patients [1]. Norman et al. conducted a retrospective review of patients ≥18 years old with OS seen between 1980 and 2019 who received at least one dose of methotrexate and showed that the median number of HDMTX doses received and number of patients who received >50% recommended doses were significantly less among patients 40 years old or older [31]. Interestingly, HDMTX AKI and associated toxicities did not vary significantly by age. Similarly, Wippel et al. conducted a single-center retrospective study that showed age over 18 years correlated with delayed methotrexate clearance and fewer overall administered doses, however, without any increased toxicity [46]. Such results suggest that clinician bias rather than outcome data may drive reductions in chemotherapy administered to older populations.

Prescribed treatment and delivered treatment are often not equivalent. In two European Osteosarcoma Intergroup trials, patients received on average 79% of the intended doxorubicin dose and 80% of the intended cisplatin dose; patients who completed only one to five of six cycles had poorer progression-free survival than those who completed all six, although the analysis did not establish a clear survival benefit from higher dose intensity [47]. A later pooled analysis found that several chemotherapy-related toxicities were associated with better survival, consistent with variation in effective drug exposure [48]. Older patients may receive a smaller proportion of the intended regimen because of organ function, toxicity, treatment delays, dose reductions, or anticipatory modification, but these data do not prove that age predicts chemotherapy benefit. Studies comparing age groups should report intended and delivered cumulative dose, relative dose intensity, delays, reductions, and toxicity.

Beyond MAP, in the first-line setting, there are few data driven systemic therapy alternatives to offer that do not extend toxicity [49] or improve outcomes [50,51]. Treatment in the relapsed–refractory setting similarly is lacking in encouraging efficacy with limited role for checkpoint inhibitor, human epidermal growth factor 2 (HER-2)-directed therapy [1], and modest overall response rate and event-free survival rates for kinase inhibitors [52,53,54], and high-dose continuous ifosfamide [55].

### 4.4. Locus of Care and Clinical Trial Enrollment

The locus of care (LOC) has also been shown to impact care delivery for patients with osteosarcoma. In their analysis of patients diagnosed with OS during 2004–2019 in the California Cancer Registry, Abrahão et al. showed that children and AYA patients treated by pediatric oncologists rather than medical oncologists were more likely to receive guideline-concordant care [34]. In the Initiative to Maximize Progress in Adolescent Cancer Therapy (IMPACT) cohort study, Mortazavi et al. sought to compare differences in demographics, disease and treatment characteristics, and survival among AYAs with osteosarcoma or Ewing sarcoma treated at adult versus pediatric centers in Ontario Canada between 1992 and 2012 [56]. Of the 137 AYA patients diagnosed with OS, those treated at a pediatric center were more likely to be enrolled in a clinical trial (55% versus just 1%) and received higher cumulative chemotherapy doses. Despite this, there were no statistical differences in EFS following multivariable analysis on the impact of LOC.

Clinical trials for osteosarcoma have historically focused on the pediatric and AYA population [7], with many studies limiting adult and elderly enrollment with age cut-offs such as 25 years old [57,58] and 40 years old [49]. Similarly, many studies have also limited pediatric enrollment, employing an 18-year-old and older barrier [53,59]. There continues to be a consistent trend of a lack of transitional clinical trials including both pediatrics and adult patients. A recent time-trend analysis of >1000 clinical trials for AYA-relevant malignancies registered on ClinicalTrials.gov from 2019 to 2023 showed that 78% were classified as adult trials with an ≥18 year-old barrier, whereas only 21% were transitional, and only 0.5% were strictly pediatrics (<18 years old) [60].

### 4.5. Is Adult Osteosarcoma Just Treated Less Effectively?

With such clear differences in disease presentation [2] and receipt of guideline-concordant care [34,56], it is easy to extrapolate why adults with osteosarcoma may have poorer outcomes as compared to their pediatric counterparts. However, we have also presented conflicting evidence this does not capture this full picture [12,13,15,56]. As such, many have sought to investigate for underlying differences between pediatric and adult osteosarcoma in the tumor biology itself.

## 5. Leveraging the Genomic Complexity of Osteosarcoma

High-grade conventional osteosarcoma is characterized by marked structural genomic instability rather than a single recurrent driver. Germline predisposition through Li–Fraumeni syndrome and hereditary retinoblastoma established the roles of *TP53* and *RB1* [61,62], while tumor profiling also identifies frequent disruption of *CDKN2A/CDKN2B* and other cell-cycle pathways [63,64,65]. Approximately one quarter of patients harbor a pathogenic germline variant in a cancer-susceptibility gene [64]. Chromothripsis, reported in 74% of cases in one recent series, can generate oncogenic rearrangements, clonal diversification, and intratumoral heterogeneity [66]. This architecture provides biological rationales for targeting checkpoint dependence, DNA-repair vulnerability, cell-cycle dysregulation, *MYC*-associated programs, and angiogenic signaling, but alteration frequency alone does not establish treatment sensitivity.

An age-associated alteration may be prognostic or simply associative; it is not evidence that a particular age group benefits more from a specific drug. Predictive inference requires biomarker-stratified treatment effects or a formal treatment-by-age interaction. The evidence summarized below is predominantly preclinical or early phase; so, the proposed age-target relationships are hypotheses for testing rather than clinically actionable findings (Table 1).

### 5.1. TP53 and WEE1 Inhibitors

Loss of *TP53* function in osteosarcoma provides a biological rationale for WEE1 inhibition. WEE1 kinase regulates the G2/M checkpoint and helps maintain genomic stability [77]; *TP53*-deficient cancers may become more dependent on this checkpoint under replication stress. Studies in other solid tumors have evaluated WEE1 inhibitors alone and in combination [78,79,80,81,82]. In the FOSTER genomic analysis, co-occurring *TP53*, *RICTOR*, and *RB1* alterations generated hypotheses for combined checkpoint and mTOR-directed strategies [63]. Avutu et al. evaluated azenosertib plus gemcitabine in 31 adult and pediatric patients aged 12–76 years with relapsed or refractory osteosarcoma; among 28 efficacy-evaluable patients, 18-week event-free survival was 39% [68]. The study was not stratified by *TP53* status or age, and no interaction between treatment and age was examined. The regimen therefore warrants further study, though nothing here indicates that younger patients, or those with *TP53* alterations, derive greater benefit.

### 5.2. RB1 and PARP Inhibitors

*RB1* alterations are reported more often in younger osteosarcoma cohorts and disrupt cell-cycle control, DNA double-strand break repair, and mitotic fidelity [69,83,84,85,86]. Preclinical work has shown selective sensitivity of *RB1*-defective cancer cells to PARP1/2 inhibition [83]. A phase II study of olaparib plus ceralasertib in recurrent osteosarcoma reported a 4-month event-free survival of 13.5% ± 5.6% and an objective response rate of 2.7% among 37 heavily pretreated patients [70,87,88]. Results were not stratified by *RB1* status, enrollment was limited to ages 12–40 years, and no age interaction was tested. The association of *RB1* alterations with younger age is therefore prognostic or descriptive, not evidence that younger patients benefit more from PARP inhibition. A broad age range at enrollment and stratification by *RB1* status would allow biomarker and age interactions to be tested directly.

### 5.3. MYC Amplification

*MYC* amplifications have been shown to be one of the most significant contributions to poor prognosis in osteosarcoma patients [89,90], with overexpression associated with higher rates of metastases and short metastasis-free survival time [7,91]. Early investigations into the efficacy of Olaparib and Ceralsertib presented at ASCO 2026 [87] have shown that therapy resistance may be associated with *MYC* amplification. Jiang et al. identified *MYC*-amplified osteosarcoma, particularly that with homologous recombination deficiency, as having a possible association with cisplatin chemoresistance [89]. With such associations with resistance and poor prognosis, stratifying future investigations by *MYC* status may be critical.

*MYC* amplification has been associated with age < 40 years and with response to ifosfamide in observational profiling [69]. Preclinical studies also link *MYC* copy number or expression to paclitaxel, mTORC1, and Aurora kinase vulnerabilities [71,72,73,92]. These studies support biomarker-driven investigation, but they do not establish an age-predictive effect. Trials of candidate *MYC*-directed strategies will need *MYC*-status stratification and enrollment across the age spectrum before they can show whether treatment effect varies independently with age.

### 5.4. CDK4/6 Inhibition

Deletion of *CDKN2A/CDKN2B* is associated with poor prognosis in osteosarcoma [89] and can increase CDK4/cyclin D activity, providing a rationale for CDK4/6 inhibition. Single-agent CDK4/6 inhibitors have shown limited activity in preclinical osteosarcoma models, whereas combined CDK4/6 and PI3K/mTOR inhibition has improved tumor control in selected models [75,93]. CDK4 and *RB1* alterations appear relatively exclusive, and *CDKN2A/CDKN2B* alterations have been reported more often in older patients [63,69]. This pattern generates a testable hypothesis for *RB1*-intact, CDK4-pathway-altered tumors; it does not show that adults benefit more from CDK4/6 inhibitors. Testing this would require prospective selection or stratification by the relevant biomarker, with age entered as a separate interaction term.

### 5.5. VEGFR2 and MET

Vascular endothelial growth factor (VEGF) contributes to angiogenesis and osteosarcoma progression [94]. VEGF-A expression and VEGFR2 signaling have been associated with metastasis and poorer outcomes [95,96]. Multikinase inhibitors that target VEGFR pathways, including regorafenib and sorafenib, have shown modest activity in recurrent metastatic osteosarcoma [53,54,59]. Cabozantinib targets VEGFR2 and *MET*; constitutively active *MET* can transform osteoblasts in experimental models [97]. In the phase II CABONE study, 12% of patients with osteosarcoma achieved an objective response at 6 months and 33% were progression-free at 6 months [52]. The ongoing phase II/III AOST2032 trial evaluates cabozantinib with frontline chemotherapy but limits enrollment to patients younger than 40 years [76]. These data support activity in an unselected population. Neither age nor VEGFR2/*MET* expression has been shown to predict cabozantinib benefit.

### 5.6. Genetic Complexity by Age

As mentioned previously, one of the biggest challenges in osteosarcoma research, drug discovery, and clinical treatment has stemmed from the fact that osteosarcoma is characterized by high genomic complexity and significant heterogeneity [98]. Considering the dramatic differences in disease outcomes between young and adult patients with osteosarcoma, investigating for differences in genetic signatures is an obvious consideration to provide some clarity to the disease’s underlying genetic complexity. Outani et al. were able to show age-associated genetic signatures with *CCNE1*, *MCL1*, *MYC*, and *RB1* alterations being significantly associated with younger age (<40 years old), while CDK4, *CDKN2A*, *CDKN2B*, *H3F3A*, *KMT2D*, *MDM2*, *RAC1*, and *SETD2* alterations significantly associated with older age (>40 years old) [69]. An analysis conducted by Zou et al. on 194 osteosarcoma patients across the age spectrum showed that mutations in those over the age of 15 yo were mainly in cell cycle and PI3K/mTOR pathways, while younger patients carried higher rates of mutations in cell cycle and angiogenesis [74]. Younger patients carried higher rates of *VEGFA*, *CCND3*, *TFEB* mutations, and older patients had higher rates of *CDKN2A/B* deletions [74]. Further work is needed to confirm these initial investigations into genomic differences by age-signature, which will be critical considering the potential therapeutic implications this may have.

The diversity in factors that drive the development of OS may explain the underlying genomic differences between pediatric and adult OS. Recent data have suggested that osteosarcomas can form at multiple points in development and from a diversity of cells, ranging from mesenchymal stem cells, osteoblasts, and dysregulated osteoclasts [99]. Pediatric osteosarcomas grow in rapidly expanding bones near growth plates, versus the relative increase in axial tumors noted in the adult population [2]. The aging bone microenvironment also likely contributes to abnormal osteoclast activity and tumorigenesis with interleukins, cytokines, and growth-related factors having been linked to osteosarcoma development [99,100,101]. Finally, differences in epigenomic programs with age may modulate the accessibility of the underlying genetic complexity characteristic of pediatric and adult osteosarcoma.

Age-associated genomic patterns can guide the design of biomarker studies, but they should not determine treatment outside a trial. The FOSTER consortium reported relative exclusivity between CDK4 and *RB1*, suggesting that CDK4/6 inhibition could be studied in *RB1*-intact, CDK4-altered tumors [63]. Likewise, the greater frequency of *RB1* alterations in younger cohorts provides a rationale for age-inclusive PARP-inhibitor studies stratified by *RB1* status [69]. Differential treatment benefit by age is not demonstrated by either observation, and both await independent validation and formal interaction testing.

## 6. Epigenetics of Osteosarcoma

Epigenetic dysregulation, including altered DNA methylation, histone modification, chromatin accessibility, and non-coding RNA programs, is implicated in osteosarcoma development and progression [102,103,104]. Direct comparisons of osteosarcoma epigenomes across prespecified age groups remain scarce.

Recent work by Lopez-Fuentes et al. has characterized osteosarcoma by epigenetically defined subtypes [104]. Following an analysis of chromatin accessibility using ATAC-seq, they identified two subtypes, “early osteoblast-derived” (EOD) cell state and “late-osteoblast-derived” (LOD) cell state characterized by upregulation in early and late bone development, respectively, with specific transcription factors that regulate the transition from one state to another in the same tumor. They further were able to validate that the two epigenetically defined cell states have differential drug response to targeted therapies with EOD cell lines responding to aurora kinase B inhibitors and LOD responding to the mitogen-activated protein kinase (MEK) inhibitor trametinib [104]. Despite what the biology may suggest, it remains untested how EOD and LOD cell states may correlate with age, and this serves as a promising mechanism to investigate some of the age-related differences in osteosarcoma that we observe.

Age-related epigenomic remodeling has been described across cancers [105], but applying those findings to osteosarcoma is an extrapolation. A relationship between age, osteoblast differentiation state, and therapeutic sensitivity remains plausible but untested. Age-linked epigenetic subtyping should therefore be treated as a research question, with validation in age-stratified osteosarcoma cohorts before clinical use.

## 7. Immune Composition

The osteosarcoma tumor microenvironment contains malignant, endothelial, stromal, and immune cells and is commonly enriched for tumor-associated macrophages, with variable and often limited T-cell infiltration [106,107,108]. Single-cell and spatial studies also identify myeloid-derived suppressor cells, regulatory and exhausted T cells, and other immunosuppressive states. This heterogeneity may help explain the limited activity of checkpoint blockade in unselected osteosarcoma [109,110], but the contribution of each compartment depends on phenotype and spatial context.

Pathology-based studies show that immune composition is more nuanced than a uniformly protumor macrophage model. In pretreatment biopsies, macrophage-associated transcriptional programs and greater tumor-associated macrophage infiltration were associated with less metastatic progression and better survival [111]. In 129 patients with localized osteosarcoma treated on ISG-OS1, CD8/TIA-1 cytotoxic lymphocyte infiltration independently correlated with survival, whereas PD-L1 expression in immune cells identified poorer outcomes among CD8-positive tumors [112]. These findings are prognostic and do not establish response to immunotherapy.

The use of chimeric antigen receptor T (CAR-T) therapy has been well established as a mainstay of treatment for many hematologic malignancies; however, its role in solid malignancies has yet to be well established. Numerous attempts have been made to use CAR-T cells targeting HER2 [113], ganglioside GD2 [114], folate receptor alpha (*FOLR1*) [115], leucine-rich repeat containing 15 (*LRRC15*) [116], B7-H3 [117], and Oncostatin-M [118], with limited evidence of efficacy beyond preclinical models at this time. Similarly, evidence for the use of antibody drug conjugates [119,120,121,122] and bispecific antibodies [123] remains limited at this time.

Aging is accompanied by systemic changes in naive and memory T-cell pools, cytotoxic function, exhaustion, and regulatory phenotypes [124,125]. Most of this evidence comes from cancers other than osteosarcoma. A 2026 single-cell transcriptomic and T-cell receptor sequencing study directly compared primary osteosarcoma from children and young patients with tumors from elderly patients and reported enrichment of pro-inflammatory and pro-angiogenic macrophage states, fewer cytotoxic CD8+ T and natural killer cells, and lower T-cell receptor diversity in older tumors [126]. This is direct age-stratified evidence, but it remains an early discovery study that requires independent validation and adjustment for tumor site, stage, treatment exposure, and primary versus secondary disease.

Age-specific immune data in osteosarcoma remain limited rather than absent. Future studies should combine standardized pathology, spatial profiling, and single-cell assays with prespecified age groups and should account for treatment exposure, metastatic site, comorbidity, and secondary disease. Clinical studies must also distinguish a prognostic immune pattern from a predictive biomarker of immunotherapy benefit.

## 8. Looking Ahead

Older age has consistently been associated with poorer osteosarcoma outcomes [2,4,5,6], although the magnitude and independence of this prognostic association vary across cohorts and endpoints. Current treatment approaches are largely derived from pediatric and AYA studies, while older adults are underrepresented and may receive modified or less intensive regimens because of comorbidity, organ function, toxicity, or clinician concern [4,31,32,33]. Such findings support the generation of age-inclusive guidelines; however, no standard of care exists explicitly for the adult population, and there is no momentum by any cooperative group to develop one.

**Figure 1:**
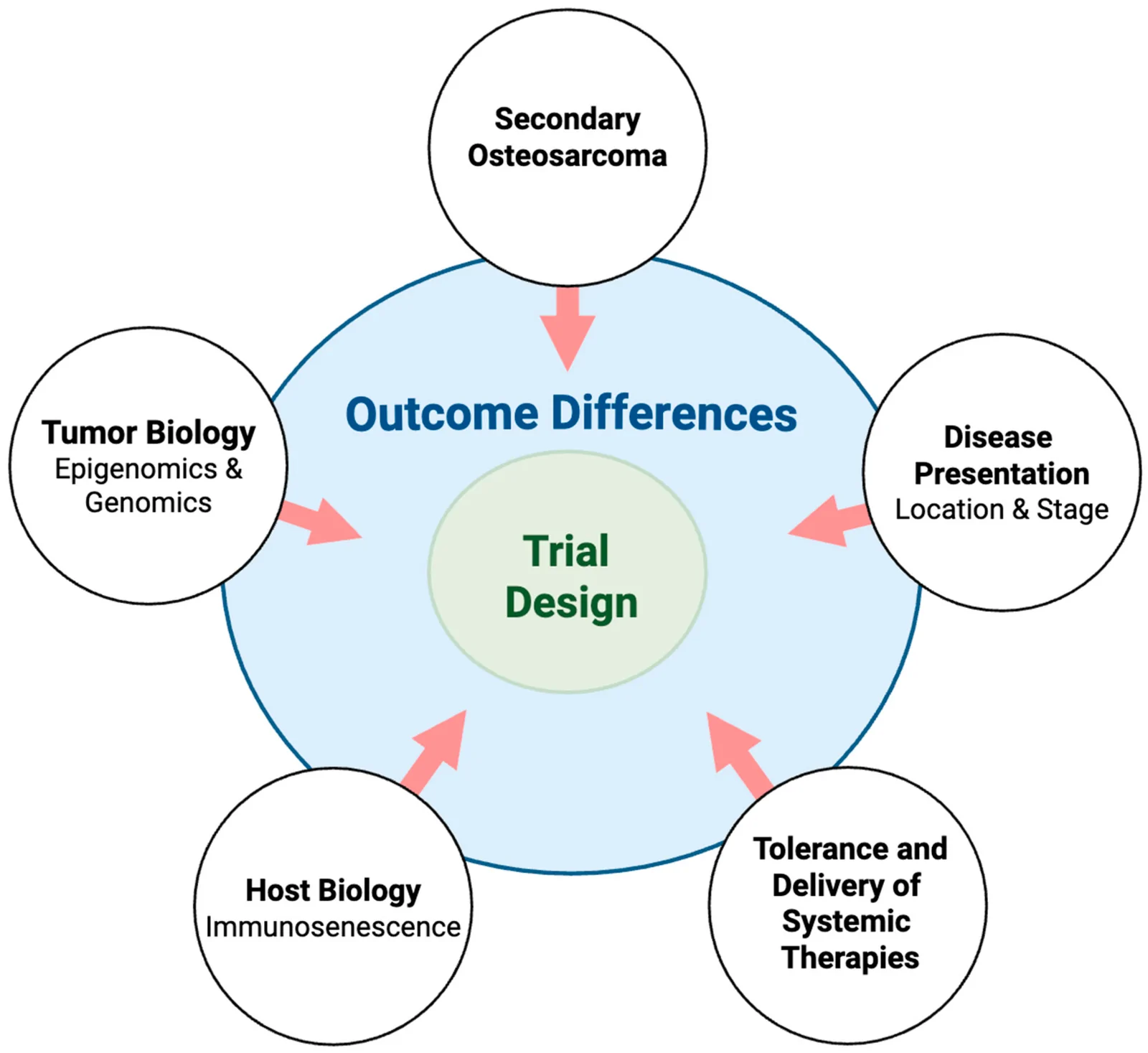
Age-dependent factors and their influence on outcome differences, and subsequently, their need to affect trial design.

We have shown age as a variable when it comes to tumor-intrinsic biology, host biology, and patients’ ability receive and tolerate treatment. Furthermore, we know that there are higher rates of secondary osteosarcoma and axial disease among adults [2], all of which indicate that adult osteosarcoma may represent a heterogenous mixture of disease states rather than a unified one. Understanding these age-associated differences, prospective studies are required to determine whether age independently predicts sensitivity to specific treatments and these variations should largely guide treatment design (Figure 1).

Trials are already incorporating predictive biomarker analyses; for example, the AOST2032 trial, investigating the use of cabozantinib with MAP in newly diagnosed osteosarcoma, aims to investigate VEGFR2, platelet-derived growth factor receptor alpha (*PDGFRA*) and C-*MET* pathway activation as potential predictive biomarkers [76]. While this represents some progress regarding the integration of biomarker data within osteosarcoma trials, such efforts remain inadequate as they continue to ignore age as a critical variable, and even further, limit outcome applicability as they are capping patient enrollment to those only under 40 years old. We should be prioritizing transitional designs and relaxing the strict age cut offs seen so often utilized in this field. Rather than exclude older patients due to fear their enrollment may affect outcome results, we suggest that future trials consider modeling age as a continuous variable while still reporting prespecified clinically meaningful age groups for interpretability.

With the explosion in our understanding of the genomic alterations driving osteosarcoma, and especially how those change with age [69,74], we recommend pooling patient data across international consortia so that any associations can be validated prospectively. Investigations should be testing treatment outcomes by both biomarker, as well as age, understanding that biomarker expression and age may often correlate. Integrating longitudinal tumor, blood, and circulating tumor DNA (ctDNA) collection will expand our understanding of how the disease responds to targeted therapies over time.

Finally, separating any variety in disease states as best as we can will help clarify predicted responses. Investigations should be adjusting for disease site, stage, histology, and resectability. Understanding that adults are not treated to the same degree as their pediatric counterparts, investigators should also stratify adult analyses by comorbidity and dose intensity, while collecting pharmacokinetic, organ-function, toxicity, and geriatric assessment data.

Until we recognize the varied and extensive impact age carries on host, disease biology, and treatment tolerance, and adjust our investigations into therapies appropriately, we fear minimal progress can be made about improving outcomes in adults with osteosarcoma.

## 9. Limitations

This narrative review has several limitations. The literature search was not conducted as a formal systematic review, and study selection and interpretation may therefore be subject to selection bias. Age definitions, endpoints, treatment eras, and adjustment strategies varied substantially across studies, limiting direct comparison. Much of the clinical evidence is retrospective, while molecular cohorts are small and may differ in tumor site, histology, primary versus secondary disease, and prior treatment. Age-specific epigenomic and immune data are particularly sparse, and several mechanistic arguments are extrapolated from preclinical models or other solid tumors. Finally, few therapeutic studies have used biomarker-stratified analyses or formal treatment-by-age interaction assessments; consequently, the review identifies hypotheses and research priorities rather than age-specific treatment recommendations.

## Figures and Tables

**Table 1:** Summary of reported age associations and associated therapeutic hypotheses. Age associations are descriptive and should not be interpreted as predictive without biomarker-stratified treatment effects or formal treatment-by-age interaction analyses.

Alteration/ Biological State	Reported Age Association	Therapeutic Hypothesis	Clinical/Preclinical Evidence	Biomarker Selection Used	Major Limitation
*TP53* mutation	<30 years old [67]	WEE1 inhibitors force cells to bypass G2/M checkpoint leading to mitotic catastrophe	Azenosertib + Gemcitabine in relapsed refractory OS, Phase I [68]	None	Did not stratify by *TP53*, did not stratify by age
*RB1* mutation	<40 years old [69]	PARPi block DNA repair commonly seen in *RB1* altered malignancy	Olaparib with Ceralasertib in recurrent unresectable OS, Phase II [70]	None	Did not stratify by *RB1* status, did not stratify by age, limited age enrollment (12–40 years old only)
*MYC* amplification	<40 years old [69]	Myc sensitized cells to taxol	Preclinical studies [71]	N/a	N/a
		MTORC1 inhibitors interrupt MTORC1 regulation of *MYC* translation	Preclinical studies [72]		
		Aurora kinase inhibitors disrupt Aurora Kinase A stabilization of *MYC* proteins	Preclinical studies [73]		
*CDKN2A/B* mutations	>40 years old [69] >25 years old [74]	Dual CDK4/6-PI3K/mTOR inhibition promotes a combination of senescence and autophagy	Preclinical studies [75]	N/a	N/a
VEGFR2/*MET* expression	<15 years old [74]	Leverage MKIs to target VEGFR2, the main receptor for *VEGFA* involved in angiogenesis, and *MET*, whose constitutively active state transforms osteoblasts to osteosarcoma	Cabozantinib + Chemotherapy in frontline setting, Phase II/III [76]	VEGFR2, *PDGFRA*, and C-*MET* pathway activation	Not planning on stratifying by age, capping enrollment at 40 years old
			Cabozantinib in advanced OS, Phase II [52]	None	Did not stratify by age
			Regorafenib in recurrent metastatic OS, Phase II [59]	Planned to stratify by age <18 or 18+ years old at inclusion	Only enrolled adult patients
			Regorafenib in recurrent metastatic OS, Phase II [54]	None	Only enrolled adult patients
			Sorafenib in recurrent metastatic OS, Phase II [53]	None	Did not stratify by age

Abbreviations: *TP53*, tumor protein p53; OS, osteosarcoma; *RB1*, Retinoblastoma 1; PARPi, poly(ADP-ribose) polymerase inhibitor; DNA, deoxyribonucleic acid; MTORC1, mammalian target of rapamycin complex 1; *CDKN2A/B*, cyclin-dependent kinase inhibitor 2A and 2B; CDK4/6i, cyclin-dependent kinase 4 and 6 inhibitors; PI3K, phosphoinositide 3-kinase; mTOR, mammalian target of rapamycin; VEGFR2, vascular endothelial growth factor receptor 2; *VEGFA*, vascular endothelial growth factor A; *PDGFRA*, platelet-derived growth factor receptor alpha.

## Data Availability

No new data were created or analyzed in this study. Data sharing is not applicable to this article.

## Abbreviations

The following abbreviations are used in this manuscript:

OS	Osteosarcoma
SEER	Surveillance, Epidemiology and End Results
RS	Relative survival
DSS	Disease-specific survival
AYA	Adolescent and young adult
EFS	Event-free survival
MAP	High-dose methotrexate, doxorubicin, and cisplatin
HDMTX	High-dose methotrexate
GCC	Guideline-concordant care
AKI	Acute kidney injury
HER-2	Human epidermal growth factor 2
LOC	Locus of care
IMPACT	Initiative to Maximize Progress in Adolescent Cancer Therapy
*TP53*	Tumor protein p53
*RB1*	Retinoblastoma 1
*CDKN2A/B*	Cyclin-dependent kinase inhibitor 2A and 2B
FOSTER	Fight Osteosarcoma Through European Research
*RICTOR*	Rapamycin-insensitive companion of mammalian target of rapamycin
PARPi	Poly(ADP-ribose) polymerase inhibitor
ASCO	American Society for Clinical Oncology
MTORC1	Mammalian target of rapamycin complex 1
CDK4	Cyclin-dependent kinase 4
CDK4/6	Cyclin-dependent kinase 4 and 6
PI3K	Phosphoinositide 3-kinase
mTOR	Mammalian target of rapamycin
VEGF	Vascular endothelial growth factor receptor
VEGF-A	Vascular endothelial growth factor A
VEGFR2	Vascular endothelial growth factor receptor 2
MKI	Mult-kinase inhibitor
DNA	Deoxyribonucleic acid
RNA	Ribonucleic acid
ATAC-seq	Assay for Transposase-Accessible Chromatin using sequencing
EOD	Early osteoblast-derived
LOD	Late osteoblast-derived
MEK	Mitogen-Activated Protein Kinase
TME	Tumor microenvironment
TAM	Tumor-associated macrophage
CAR-T	Chimeric antigen receptor T
*FOLR1*	Folate receptor alpha
*LRRC15*	Leucine-rich repeat containing 15
ICI	Immune checkpoint inhibitor
*PDGFRA*	Platelet-derived growth factor receptor alpha
ctDNA	Circulating tumor DNA
